# A comparison study between periosteum and resorbable collagen membrane on iliac block bone graft resorption in the rabbit calvarium

**DOI:** 10.1186/1746-160X-10-15

**Published:** 2014-05-10

**Authors:** Ji-Woong Yang, Hong-Ju Park, Kil-Hwa Yoo, Kwang Chung, Seunggon Jung, Hee-Kyun Oh, Hyung-Seok Kim, Min-Suk Kook

**Affiliations:** 1 Department of Oral and Maxillofacial Surgery, Chonnam National University School of Dentistry, 77 Yongbong-Dong Buk-Gu 500-757, Gwang-Ju, Republic of Korea; 2 Department of Forensic Medicine, Chonnam National University Medical School, Gwang-Ju, Republic of Korea

## Abstract

**Background:**

To compare the different resorption patterns between resorbable membrane barrier and periosteum after iliac block bone grafting radiographically and histologically.

**Methods:**

Eighteen mature male rabbits weighing from 2.0 to 2.5 kg were used. The recipient site was the rabbit skull, and autogenous iliac bone was used as the grafting material. The harvested iliac block bones were divided in the following groups: autogenous iliac block bone with preservation of the periosteum (the periosteum group), autogenous iliac block bone covered with a resorbable collagen membrane (Biomesh®, Samyang Co, Korea) after removing the periosteum (the collagen membrane group), and autogenous iliac block bones with removal of the periosteum (the control group). In each experimental group, periosteum or resorbable collagen membrane of the donor site was fixed directed to the periosteum of the recipient site. The specimens were examined macroscopically, radiographically, histologically, and histomorphometrically at every 2, 4, and 8 weeks.

**Results:**

All groups presented excellent bone graft healing state without inflammation, dehiscence, or displacement. The radiolucency increased from mild to moderate in all groups over the experiment. The mean thickness of the upper end of the cortical iliac bone graft was statistically significantly different between the control group and the periosteum group, between the four-week and eight-week control group, and between the four- week and eight-week periosteum group (p & 0.05).

**Conclusion:**

This study suggests that both the periosteum and the resorbable collagen membrane may help to prevent soft tissue infiltration into the bone graft and to reduce bone graft resorption compared to block graft alone.

## Introduction

Several alternatives for bones, such as autograft, xenograft, and other bone materials are used to treat bone destruction or bone defects caused by various diseases. Dental clinics generally perform implant installation procedures to restore the masticatory function, to increase the mean life expectancy, and to improve the quality of life. At this time, various studies are being performed to increase the volume of defective alveolar bones. Among these techniques, bone grafting is the most widely used, and autografts, allografts, xenografts and alloplasts are currently used as grafting materials [1]. Although there are increasing interests in xenografts and other alternative grafting materials, autografts are excellent grafting materials compared to other materials considering their function, form, and adaptability.

Autogenous bone materials induce osteogenesis, osteoinduction, and osteoconduction. Autogenous grafts can be cancellous, nonvascularized cortical, or vascularized cortical. Autogenous bone graft remains the most effective grafting material because it provides the three elements required for bone regeneration: osteoconduction, osteoinduction, and osteogenic cells which can regenerate new bone for oneself. Autogenous bone graft provides these three components to a limited extent as well and also provides the structural integrity important in reconstruction of larger defects. In addition, autografts can be referred as ideal grafting materials since there is rapid healing without immunological rejection [1,2].

However, autografts have several limitations such as size, shape, quantity, quality of harvested bone, and the need for additional surgery for the donor site due to complications and osteoclastic bone resorption after grafting. It is reported that onlay bone grafting using iliac bone shows bone resorption up to 50% in long-term tracking observation [3,4].

The periosteum has been studied to minimize bone resorption and increase survival rates of grafted tissues after autogenous bone graft. In 1912, Macewen [5] examined the periosteum, and Tonna [6] was the first to describe that the periosteum is composed of inner and outer layers. Fraccari and Gotte [7] reported that conservation of the periosteum instead of its removal helps in new bone formation. Conservation of the periosteum induced complete regeneration of the resected bone after hemimnadibulectomy. Tornberg and Bassett [8] reported new bone formation induced by the periosteum of an immature bone in the rat radius and ulna. In 1981, Barro and Latham [9] demonstrated more new bone formation within the periosteum by fluorescence microscopy which showed a proliferative cellular response of inner periosteal layer and renewed osteogenic activity.

Periosteum is a connective tissue membrane consisting of two layers: the inner and the outer layers The inner layer, which is close to the surface of bone, contains more cellular components than the outer layer [6]. The cellular components of the periosteum generally include cells that are responsible for bone remodeling and their precursor cells. The periosteum has a rich blood and nerve supply because many microvessels and nerves are distributed in the periosteum. The outer layer of the periosteum has more fibrous components and Sharpey’s fibers, which originate from the outer layer and reach the surface of the cortical bone through the periosteum.

Several studies of the periosteum have been performed. The bone formation of the periosteum was observed after grafting the harvested periosteum to the soft tissue [10]. The effect of periosteum on bone formation after bone grafting [11], the influence of periosteum on bone defect healing after trauma [7,12] have been reported. However, only a few studies have examined the effect of periosteum on bone graft resorption and graft survival. Therefore, it is important to study the effect of the periosteum attached to the bone graft on bone graft healing.

Additionally, GBR(guided bone regeneration), is performed frequently along with grafting for bone repair and regeneration. In this study, a protective shield and a membrane barrier were used. There are two methods of onlay bone graft, using membrane and grafting in form of block bone. In using membrane technique, the bone generation is achievable by using GBR in the bone loss area, and the volume of absorbed alveolar bone can be enhanced so that implant materials can be successfully implemented. Various studies proved the importance of the stability of membrane, the hole size of membrane, and sealing performance of margin [13-15].

The characteristics of membrane barriers are biocompatibility, tissue integration, space maintenance, and easy handling [12,16,17]. The membrane barrier decreases osteoclastic bone resorption and increases new bone formation by preventing invasion of undesirable cells which have no osteogenic capacity into the bone defect region during its repair [18,19]. The membrane barriers can be divided into the resorbable and non-resorbable types. Non-resorbable membrane barriers were used in clinical practice for the first time. Since the non-resorbable membrane barrier is a biologically inert material and hence it does not dissolve *in vivo*, a second surgery is required for its removal, which is associated with complications [20,21]. On the other hand, the resorbable membrane barrier maintains its shape and barrier function during certain period of time. This type of membrane barrier does not require a second surgery for its removal.

Though using non-absorbable membrane enhances the possibility of bone regeneration, the membrane exposure can cause bacterial infection, and the inflammation response in early stage can bring out membrane removal.

There have been plenty of researches on the resorbable membrane barrier but not on comparative study between the resorbable membrane barrier and periosteum after autogenous bone grafting. We conducted a comparative study to evaluate radiographically and histologically the different resorption pattern between resorbable membrane barrier and periosteum after onlay bone grafting.

## Materials and methods

### Animals

Eighteen mature male rabbits weighing from 2.12 to 2.43 kg (mean weight, 2.23 kg) were used. The recipient site was the rabbit skull, and autogenous iliac bone was used as the grafting material. The animal care and experimental procedures were performed in accordance with the Guidelines for Animal Experimentation of Chonnam National University Hospital.

### Methods

**Figure 1:**
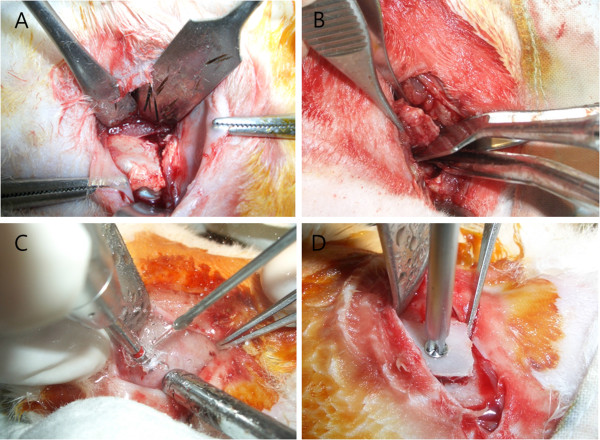
**Photographs in the experiment. A**, **B**. 10 mm (L) × 8 mm (W) × 4 mm (H) sized iliac block bones were harvested using an osteotome. **C**, **D**. The grafted bone was fixed with 6 mm miniscrews.

The rabbits were placed under general anesthesia by injecting 10 mg/kg of Rompun® (Rompun®, Bayer Co, Germany) and 200 mg/kg of Zoletil® (Zoletil®, Vibac Laboratories, France) intramuscularly, and all hair on the skull and bilateral iliac areas were removed using an electric shaver. 1:100,000 epinephrine including 2% of lidocaine was then injected, and the area was steralized with potadine. The iliac bone was exposed, leaving the periosteum intact by performing a skin incision and dissection of the bilateral iliac areas. Iliac block bones, measuring 10 mm (L) × 8 mm (W) × 4 mm (H) in size, were harvested using an osteotome (Figure 1). At that time, some of the iliac block bones were harvested with the periosteum attached to the the iliac bone, whereas the remaining iliac block bones were harvested without the periosteum by elevating it. Rabbit skulls were exposed by a midline incision along the sagittal suture, and the periosteum was elevated for placing the bone graft. Six holes were made using a round bur to induce bleeding in the grafted skulls.

**Figure 2:**
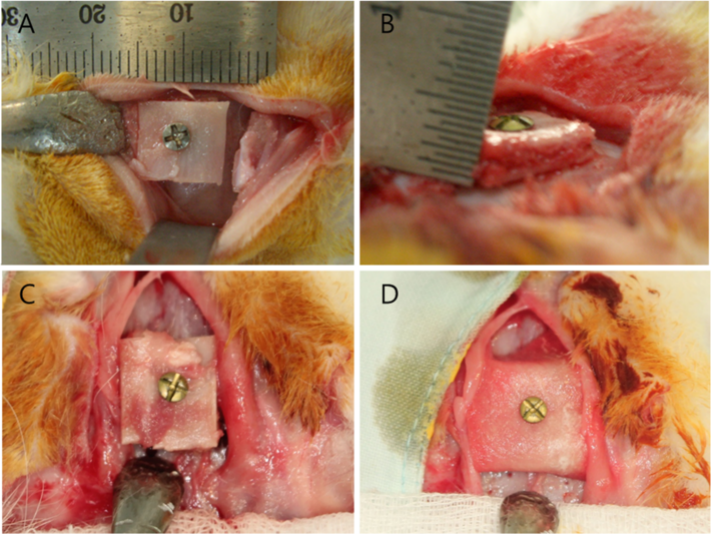
**Photographs after fixation. A**, **B**. The iliac block bone without periosteum was fixed with 6 mm miniscrews. **C**. The iliac block bone with periosteum. **D**. The iliac block bone was covered with membrane.

Subsequently, the bone grafts were placed between the skull, and the periosteum were fixed with 6 mm miniscrews (14-AT-004, *Jeil Medical Corporation*, Korea), and thus the heads of the screws were in contact with the bone graft (Figure 2). The harvested iliac block bones were divided in the following groups: autogenous iliac block bone with preservation of the periosteum (the periosteum group), autogenous iliac block bone covered with a resorbable collagen membrane (Biomesh®, Samyang Co, Korea) after removing the periosteum (the collagen membrane group), and autogenous iliac block bones with removal of the periosteum (the control group). In each experimental group, periosteum or resorbable collagen membrane of the donor site was fixed, directed to the periosteum of the recipient site.

After irrigating the surgical site by a normal saline solution, the recipient and donor sites were sutured with 3–0 Vicryl® (Vicryl®, Ethicon Co., Livingston, UK), and the skin was sutured with 3–0 Mersilk® (Mersilk®, Ethicon Co., Livingston, UK). After suturing, performing wound dressing with potadine, and confirming that the animals had awakened from anesthesia, they were shifted to the experimental animal laboratory. After surgery, a prophylactic antibiotic (Fortimicin®, Young Jin Pharm Co, Korea) and an anti-inflammatory drug (Fenaca®, Hana Pharm Co, Korea) were administered once a day for five days to prevent infection and reduce the level of pain.

The rabbits were euthanized by collecting around block bone of calvaria in experimental animal and administering 2 meq/kg KCl (KCl-20®, Daihan Co, Korea) to the ear of rabbit intravenously.

### Examination

After bone grafting and sacrificing six rabbits at every 2, 4, and 8 weeks, the grafted skulls and iliac bone grafts were resected. Macroscopic examination was done to check if the experimental condition meet the requirements. That is to say, the examination is conducted to confirm if there is any factor which can affect the result of experiments; for example, whether the graft site has inflammation, dehiscence, and displacement or absorbable membrane is well sustained.

The iliac bone graft was resected from the rabbit's skull and radiographs were taken. The size of the radiographic film was 25 cm from the edge of the X-ray tube to the rabbit's skull, and the tube voltage was 70 kVP with a 1.5 second exposure. The radiological finding was useful to check whether the shape of early graft are well maintained, to evaluate the rarefaction of cortical bone, and check the marginal loss.

The resected tissues were kept in formalin for two days and seceded in an EDTA solution. After paraffin-embedding, 5 μm tissue sections were prepared and dyed with hematoxylin and eosin before observed by optical microscopy.

The height of the upper end of the cortical bone graft was measured at 3 regions (anterior, middle, posterior) using an automated image analysis system (Aperio’s scanoscope systems®, Aperio Technologies Inc, USA).

### Statistical analysis

The data from the histomorphometric measurements were analyzed using an Independent Samples *T test* and One-way *ANOVA* for two independent samples using by *SPSS 18.0* (Chicago, IL, USA) (Table 1).

**Table 1:** Mean thickness of the upper end of the cortical iliac block bone graft among the control, periosteum, and membrane group at 2, 4, and 8 weeks (mm)

	**Control group (Mean ± SD)**	**Duncan**	**Periosteum group (Mean ± SD)**	**Duncan**	**Membrane group (Mean ± SD)**	**Duncan**
2 weeks	1.29 ± 0.02	A	1.29 ± 0.01	A	1.30 ± 0.01	A
		*				
4 weeks	0.40 ± 0.01	A	1.00 ± 0.01	B	1.02 ± 0.01	B
			**			
		†		††		
8 weeks	0.48 ± 0.02	A	0.84 ± 0.01	B	0.71 ± 0.01	C
			∮			

(p-value) * = 0.035, ** = 0.031, † = 0.038 , †† = 0.044 , ∮ = 0.023.

## Results

### Gross findings

The macroscopic examination revealed excellent bone graft healing state without inflammation, dehiscence, or displacement. The iliac bone grafted to the skull was well maintained under the lower portion of the periosteum in the control group, periosteum group, and collagen membrane group. The size of the iliac block bone graft in the control group decreased over time, but a similar volume of iliac block bone graft was observed in the periosteum and collagen membrane groups.

### Radiological findings

**Figure 3:**
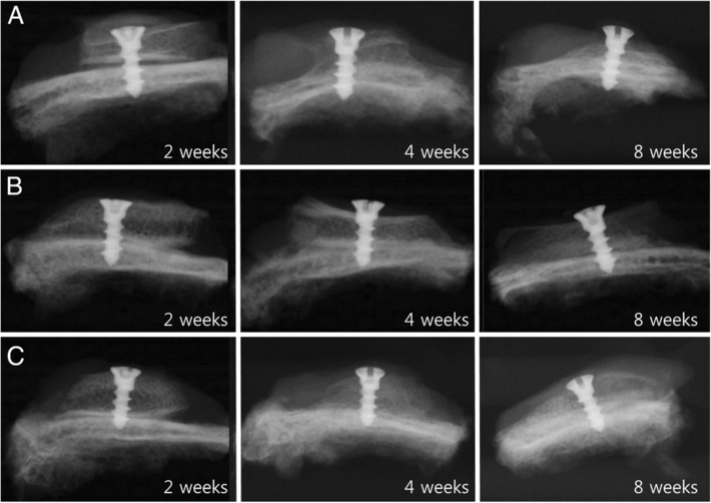
**Radiographs of each group at 2, 4, and 8 weeks after bone graft. A**. Control group, **B**. Periosteum group, **C**. Membrane group.

The radiolucency increased from mild to moderate in all groups over experiment. At 8 weeks, the initial shape of the iliac block bone graft was not observed because the iliac block bone graft, which showed maximum radiolucency, was resorbed. Generally, even if the height of iliac block bone graft was less, its original shape was maintained, but minimum iliac block bone graft resorption was seen in all groups. Mild radiolucency was observed in the collagen membrane group, but the overall shape of the iliac block bone graft was maintained (Figure 3).

### Histological findings

#### Control group

##### Two weeks

The iliac block bone graft was in close contact with the periosteum and the skull, and soft tissue ingrowths were observed. Inflammatory cell infiltration was not observed around the iliac block bone graft, but new bone formation was observed around the iliac block bone graft. Iliac block bone graft resorption by osteoclasts was not observed.

##### Four weeks

Compared to the two-week control group, overall resorbed pattern of the upper iliac block bone graft and immature new bone formation were observed. The boundary of the irregular cortical bone was observed along with the periosteum adjacent to iliac block bone graft, and osteoclast activation was observed in the area where the skull was in contact with the iliac block bone graft. The activation of osteoblasts was negligible.

##### Eight weeks

**Figure 4:**
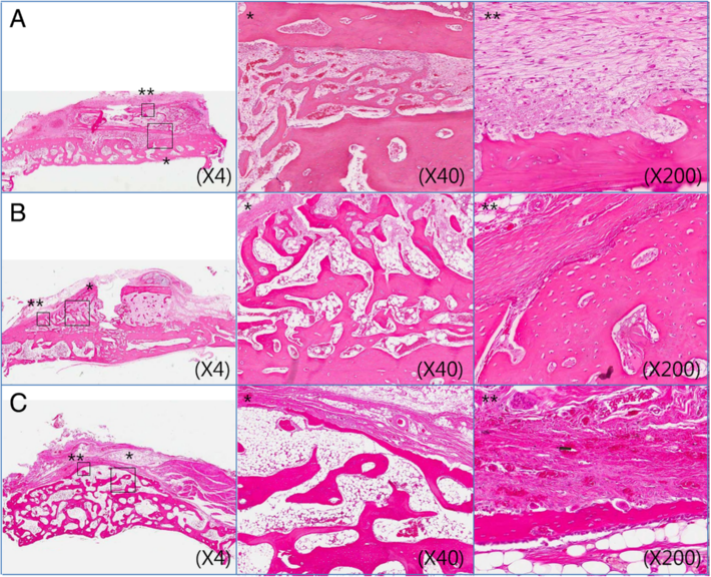
**Photomicrographs of control group (H & E). A**. At 2 weeks, **B**. At 4 weeks, **C**. At 8 weeks after bone graft. * means 40 magnification, ** means 200 magnification.

The iliac block bone graft did not show its original shape because it was widely resorbed at the boundary of the periosteum. Soft tissue ingrowth and fibrous cells were observed. The decrease in thickness due to the resorption of cortical bone, and remodeled bone were observed within the marrow. The activation of osteoblasts around the iliac block bone graft was negligible, and resorption of iliac block bone graft by osteoclasts was observed (Figure 4).

#### Periosteum group

##### Two weeks

The iliac block bone graft was in close contact with the periosteum and the skull. No inflammatory cell infiltration was observed around the iliac block bone graft, and soft tissue ingrowths were observed. Immature new bone and periosteum of the iliac block bone, a distinct margin between the periosteum of the donor site and that of the recipient site, and no revascularization was observed at the site contacted to calvaria. Resorption of the iliac block bone graft by osteoclasts was not observed.

##### Four weeks

The iliac block bone graft and the skull were well connected by the newly formed bone and by the activation of osteoblasts, little osteoclastic activity in the upper part of the iliac block bone graft, and mild osteoclastic bone resorption were observed. The periosteum of the iliac block bone graft was in close contact with the periosteum of the skull, and hence it was difficult to distinguish the periosteum of the recipient site from the periosteum of iliac block bone graft.

##### Eight weeks

**Figure 5:**
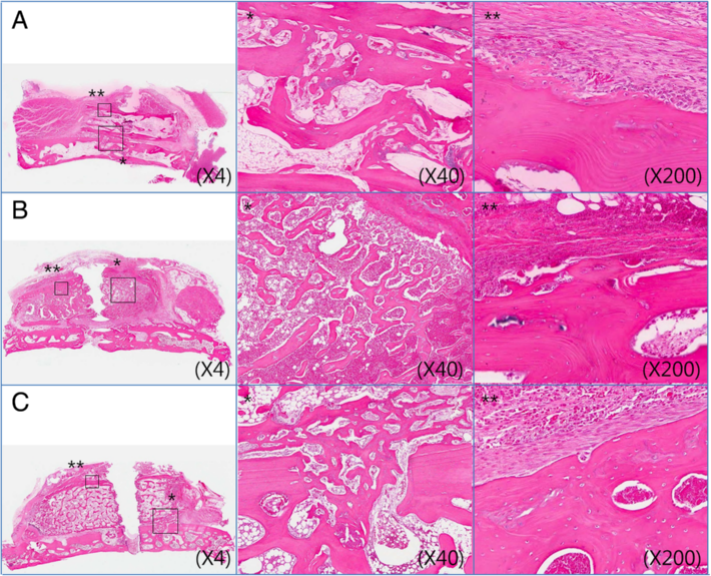
**Photomicrographs of periosteum group (H & E). A**. At 2 weeks, **B**. At 4 weeks, **C**. At 8 weeks after bone graft. * means 40 magnification, ** means 200 magnification.

The height of the iliac block bone graft was generally lesser, but the original shape of the iliac block bone graft was maintained. The iliac block bone graft was in close contact with the skull, and the activation of osteoblasts and bone remodeling were progressing continuously. Mature marrow was observed in the iliac block bone graft. The boundary between the periosteum of the skull and the periosteum of the iliac block bone graft was unclear, and little osteoclastic activity in the adjacent areas and mild irregular iliac block bone graft resorption were observed (Figure 5).

#### Collagen membrane group

##### Two weeks

The iliac block bone graft was in close contact with the periosteum and the skull. No inflammatory cell infiltration was observed around the iliac block bone graft, and upper part of the resorbable collagen membrane was observed. Immature, newly formed bone was observed in the adjacent area of the skull, no resorption of the iliac block bone graft by osteoclasts was observed, and a clear boundary was observed between the resorbable collagen membrane and the periosteum of the skull.

##### Four weeks

Compared to the collagen membrane group at two-week, the overall shape of the iliac block bone graft was well maintained and resorbable collagen membrane was observed.

An unclear boundary between the resorbable collagen membrane and the periosteum of the skull could be observed. The iliac block bone graft and the skull were well connected by the newly formed bone, and the activation of osteoblasts was observed.

Little osteoblastic activity, osteoclast activation, irregular boundary of cortical bone, and osteoclastic bone graft resorption were observed below the membrane barrier.

##### Eight weeks

**Figure 6:**
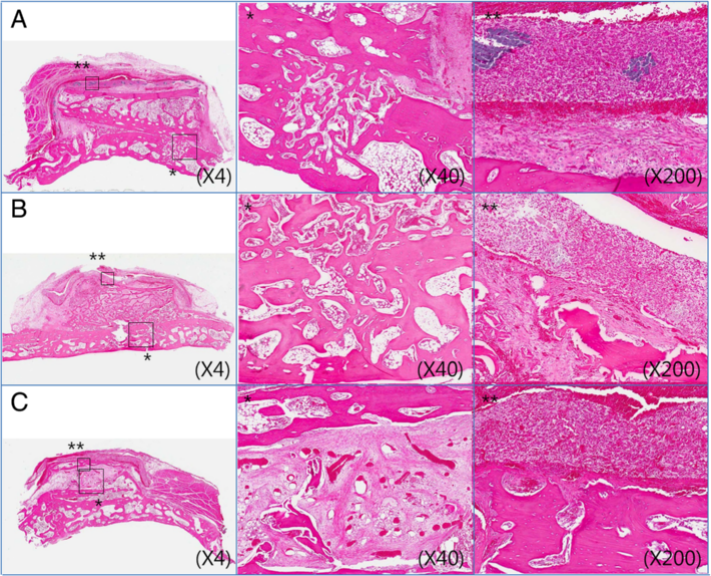
**Photomicrographs of collagen membrane group (H & E). A**. At 2 weeks, **B**. At 4 weeks, **C**. At 8 weeks after bone graft. * means 40 magnification, ** means 200 magnification.

The resorbable collagen membrane covering the iliac block bone graft did not maintain its continuity, and the graft showed a partial resorption pattern. The height of iliac block bone graft was generally lesser, and resorption of the grafted bone at both superior margins was observed. The iliac block bone graft was incorporated into the skull. In addition, continuous osteoblastic activation and bone remodeling, as well as osteoclast activation and bone remodeling were observed around the periosteum of the skull (Figure 6).

### Histomorphometric findings

The mean thickness of the upper end of the cortical iliac bone graft displayed statistically significant difference between the control group and the periosteum group at four-week (p = 0.035) and eight-week (p = 0.038), between the control group and the membrane group at four-week (p = 0.031) and eight-week (p = 0.023), and between the periosteum group and the membrane group at eight-week (p = 0.044). The Duncan test for a post-hoc comparison of the *ANOVA* analysis showed a significant difference in the mean thickness of the upper end of the cortical iliac bone graft among the control group, periosteum group, and collagen membrane group at eight-week (Table 1).

## Discussion

The autograft is considered as a gold standard of grafting materials, and it is the only available grafting material for clinical use that can lead to bone formation [2]. Hislop *et al*[22] reported that autografts show high performance in bone formation, and they fulfill most of the requirements for dental implants.

Endochondral bones, such as the rib, iliac bone, and tibia, show increased osteoclastic bone resorption than the membranous bones, such as mandibular symphysis and the ascending ramus. The study by Koole *et al*[23] demonstrated that the iliac crest bones undergo more resorption than the mandibular symphysis bones. For these reasons, iliac bone was selected due to its high resorption rate.

There are various factors that affect new bone formation. Moseley [24] reported that these factors include conservation of marrow and periosteum and components of new bone. Even though they are considered to be the most effective grafting materials, autografts have the largest drawback, that is to say osteoclastic bone resorption after surgery. This study pays particular attention to the components and bone formation function of the periosteum to reduce osteoclastic bone resorption.

An initial study of the periosteum was performed by Macewen [5] in 1912. Since then, studies assessing the effect of the periosteum on bone formation have been performed regularly, and several researches have confirmed the importance of the periosteum.

Ilizarov and Schreiner [25] emphasized the importance of conservation of the periosteum and inner layer of the periosteum through an experiment in dogs, and Kawamura *et al*[26] proved that the periosteum is involved in new bone formation. Cohen and Lacroix [27] reported that bone formation is affected by the degree of surgical damage to the periosteum and the form of the periosteum while harvesting it. Fraccari and Gotte [7] reported that the periosteum contributes to bone remodeling in hemimandibular defects. In addition, Tornberg and Bassett [8] reported that periosteum of immature bone induces bone formation in case of rat radius and ulna.

Narang and Laskin [28] stated that bone formation will not occur in the absence of periosteum, and the bone would be replaced by fibrous tissue. Barro and Latham [9], and Burstein *et al*[29] used fluorescence microscopy to examine the differences between healing of bone with periosteum and healing of bone without periosteum, and they reported that more new bone formation occurred around the periosteum. On the other hand, the periosteum plays an important role in bone remodeling by helping in bone formation and resorption, and it also plays a role in osteoclastic bone resorption since it contains osteoblasts and osteoclasts as well as osteogenic precursor cells in the inner layer [30].

Widmark *et al*[31]. and Bruggenkate *et al*[32]. reported that autografts undergo substantial osteoclastic bone resorption which was already obvious after 4 months (25%). Several studies, except for one study of the periosteum, were conducted to prevent this occurrence. The concept of using a membrane barrier on bone graft is appealing. Buser *et al*[33]. reported that it is possible to reduce the resorption of autograft by using an e-PTFE (expanded polytetrafluoroethylene) membrane barrier or collagen membrane.

The membrane barrier reduces osteoclastic bone resorption and prevents undesirable cell invasion into the bone defect during its repair [18,19]. The membrane barrier should have characteristics such as biocompatibility, tissue integration, cell occlusivity, space maintenance, stability, ease of use, and adjustable biodegradation [12,16,17]. Membrane barriers can be divided into the resorbable and non-resorbable types. The membrane barrier that was used in clinical practice for the first time was of the nonresorbable type and displayed several disadvantages, such as difficulty in use, dehiscence, infection, and the need for second surgery for its removal. Therefore nowadays, clinicians and researchers use a resorbable membrane barrier.

In a study of a resorbable membrane, Dahlin *et al*[34] reported that a resorbable membrane can prevent the invasion of the connective tissue into the osseous defects. Linde *et al*[35] reported that a resorbable membrane also enhances bone formation at the cellular and molecular level. Takata *et al*[36] also reported that a resorbable membrane promotes new bone formation during guided bone generation. Resobable membranes composed of lactic acid, glycolic acid polymers, lactide/glycolide copolymers, or collagen are developed and utilized [37-40], and resorbable membranes are degraded into small particles before the complete metabolization, being harmless to hemans.

Carpio *et al*[41] reported that it is possible to overcome the drawbacks of a membrane barrier by using a resorbable collagen membrane. Collagen membrane barriers reduce the risk of flap dehiscence, and infection because the barrier is better organized and easy to use. A resorbable membrane barrier maintains its shape and function for a certain period of time, and it also does not need a second surgery for its removal. On the other hand, a certain degree of robustness of the resorbable membrane barrier is required until a satisfactory level of healing and regeneration is achieved.

Neither localized tissue reaction nor systemic reaction of intermediate by-products of the resorbable membrane should be in the degradation process. An autograft resorbs over time [42-45]. Bruggenkate [32] reported a 50% osteoclastic resorption of mandibular symphysis grafts, and Widmark *et al*[31] reported a 60% osteoclastic resorption of chin grafts in 1 year. Jardini *et al*[45] reported a 24% osteoclastic bone graft resorption in 45 days in an experiment in calvarial bone graft into the mandibular angle. This study showed that revascularization occurs as a result of resorption of the cortical bone graft by osteoclasts and growth of blood vessels. Hammack and Enneking [46] reported that such vascularization occurs 6 days after bone grafting, and complete revascularization is achieved within 1 to 2 months after bone grafting.

This study did not demonstrate osteoclastic bone graft resorption in the histology findings at two weeks after bone grafting. On the other hand, the cortical bone graft which was close to the periosteum was generally resorbed by osteoclasts within 4 to 8 weeks after bone grafting. The bone graft was incorporated into the bone. The two-week periosteum group showed that the periosteum of the bone graft was in close contact with the periosteum of the skull, but revascularization between the periosteum of the bone graft and periosteum of the skull was not observed. In the four-week periosteum group, the periosteum that was attached to the bone graft was in close contact with the periosteum of the skull, and union between the periosteum at the recipient site and the periosteum of bone graft was observed. Regarding revascularization of the bone graft, previous studies have shown that this process did not occur concurrently with cortical bone graft resorption by osteoclasts; however, a close contact between the periosteum of bone graft and the periosteum of the recipient site contributes to the revascularization of the periosteum thus results in that of the bone graft.

The two-week collagen membrane group showed that the resorbable collagen membrane was observed in the upper part of the graft, and a clear boundary with revascularization was not observed between the resorbable collagen membrane and the periosteum of the recipient site. On the other hand, in the eight-week collagen membrane group, the continuity of the resorbable collagen membrane was not maintained. Patterns of partial bone graft resorption, irregular boundary of the cortical bone graft. and osteoclastic bone resorption were observed along with the osteoclastic activity in the upper part of the bone graft. There was no inflammatory cell invasion or immune rejection during the entire period. The resorbable collagen membrane may decrease the activation of osteoclasts and osteoclastic bone graft resorption based on the result that the part of the resorbed membrane barrier showed more osteoclastic bone graft resorption and osteoclasts than the part of the non-resorbed membrane barrier. In addition, the level of osteoclastic bone graft resorption was lower than that in the control group.

Compared to the control group, osteoclasts were not observed in the collagen membrane or the periosteum group. After bone grafting, osteoclasts originated from the recipient site in the control group. This result suggests that the periosteum and the resorbable collagen membrane attached to the bone graft inhibit osteoclasts that originate from the recipient site.

A resorbable collagen membrane did not cause infiltration of inflammatory cells, or tissue reaction, and it played the role of inhibiting the osteoclasts before bone graftresorption occurred. On the other hand, the periosteum not only acts as a continuous physical barrier, but also as an activator of revascularization by close contact with the periosteum of the recipient site. It represents that the periosteum helps revascularization of the bone graft.

These results suggest that the periosteum and the resorbable collagen membrane attached to the bone graft decrease osteoclastic bone graft resorption caused by soft tissue ingrowths. In particular, the periosteum plays a role in revascularization of the bone graft in the early stage. The reduction of bone resoprtion rate and more prompt engraftment can be obtained by grafting block bone with bone membrane to edentulous patient who suffered large bone defect from fracture and absorption of alveolar ridge.

If harvesting of the iliac block bone with the periosteum is performed, besides several side effects, such as the complexities of surgery and increased operation time, negative influences, such as more damage to the surrounding tissue and decreased donor site healing, must be compared with those of harvesting of the iliac block bone without the periosteum. Therefore, the use of a membrane barrier to reduce osteoclastic bone graft resorption is a good alternative if there is no time to harvest the bone and to preserve the periosteum during surgery. Several studies [47,48] have used mineralizing substances to promote bone regeneration under the resorbable collagen membranes, and substances that promote bone regeneration have also been reported. Hence, some good results are expected.

Furthermore, additional experiments and analyses of new membrane barrier materials and substances that minimize osteoclastic bone graft resorption and promote bone regeneration will be needed in the future.

## Conclusion

This study suggests that both the periosteum and the resorbable collagen membrane may help to prevent soft tissue infiltration into the bone graft and to reduce bone graft resorption compare to block graft alone.

## Competing interests

The authors declare that they have no competing interests.

## Authors’ contributions

JW Yang and HJ Park participated in the design of the study, analyzed the data, and made a draft of the manuscript. KH Yoo revised the manuscript. K Chung carried out collected the data. SG Jung analyzed the data. HK Oh participated in the sequence alignment and coordination. HS Kim performed the pathological analysis and statistical analysis. MS Kook participated in the design of the study, carried out the animal experiments, analyzed the data, and revised the manuscript. All authors read and approved the final manuscript.
